# Sex‐gender disparities in nonagenarians with acute coronary syndrome

**DOI:** 10.1002/clc.23545

**Published:** 2021-01-19

**Authors:** Pedro L. Cepas‐Guillen, Julio Echarte‐Morales, Eduardo Flores‐Umanzor, Andrea Fernandez‐Valledor, Guillem Caldentey, Ana Viana‐Tejedor, Eduardo Martinez Gomez, Elena Tundidor‐Sanz, Javier Borrego‐Rodriguez, Pablo Vidal, Marc Llagostera, Xavier Quiroga, Xavier Freixa, Felipe Fernández‐Vázquez, Manel Sabate

**Affiliations:** ^1^ Cardiology Department Cardiovascular Institute (ICCV), Hospital Clinic, IDIBAPS, University of Barcelona Barcelona Spain; ^2^ Cardiology Department Complejo Asistencial Universitario de León León Spain; ^3^ Cardiology Department Hospital del Mar Barcelona Spain; ^4^ Cardiology Department Hospital Universitario Clínico San Carlos Madrid Spain

**Keywords:** acute coronary syndrome, elderly, myocardial infarction, ST‐segment elevation myocardial infarction, women

## Abstract

**Background:**

Acute coronary syndrome (ACS) remains one of the leading causes of mortality for women, increasing with age. There is an unmet need regarding this condition in a fast‐growing and predominantly female population, such as nonagenarians.

**Hypothesis:**

Our aim is to compare sex‐based differences in ACS management and long‐term clinical outcomes between women and men in a cohort of nonagenarians.

**Methods:**

We included consecutive nonagenarian patients with ACS admitted at four academic centers between 2005 and 2018. The study was approved by the Ethics Committee of each center.

**Results:**

A total of 680 nonagenarians were included (59% females). Of them, 373 (55%) patients presented with non‐ST‐segment elevation ACS and 307 (45%) with ST‐segment elevation myocardial infarction (STEMI). Men presented a higher disease burden compared to women. Conversely, women were frailer with higher disability and severe cognitive impairment. In the STEMI group, women were less likely than men to undergo percutaneous coronary intervention (PCI) (60% vs. 45%; *p* = .01). Overall mortality rates were similar in both groups but PCI survival benefit at 1‐year was greater in women compared to their male counterparts (82% vs. 68%; *p* = .008), persisting after sensitivity analyses using propensity‐score matching (80% vs. 64%; *p* = .03).

**Conclusion:**

Sex‐gender disparities have been observed in nonagenarians. Despite receiving less often invasive approaches, women showed better clinical outcomes. Our finding may help increase awareness and reduce the current gender gap in ACS management at any age.

## 1 INTRODUCTION

Over the coming years we shall witness a progressive increase in the aging of the population that will lead to several social and medical challenges. In the field of cardiovascular diseases, aging causes a significant clinical heterogeneity in which sex differences play a key role. Acute coronary syndrome (ACS) remains one of the leading causes of morbidity and mortality for women, and increases with age. Nevertheless, older women continue to be underrepresented in clinical trials and their management and outcomes are underexplored.^1^ This fact is even more relevant in patients with extreme age, such as nonagenarians, who are usually excluded from the main clinical trials because a prolonged follow‐up may be compromised by limited life expectancy. However, the nonagenarian population will become clinically and numerically relevant in our daily routine practice in the near future and data about their prognosis in ACS context is scarce, especially in women. It is expected that female life expectancy will break the 90‐year barrier by 2030, a level that was deemed unattainable by some at the beginning of the 21st century.^2^ Therefore, there is an unmet need regarding ACS in this fast‐growing and predominantly female population. The aim of this study was to compare sex‐based differences in ACS management and long‐term clinical outcomes between women and men in a cohort of nonagenarians.

## 2 METHODS

**FIGURE 1:**
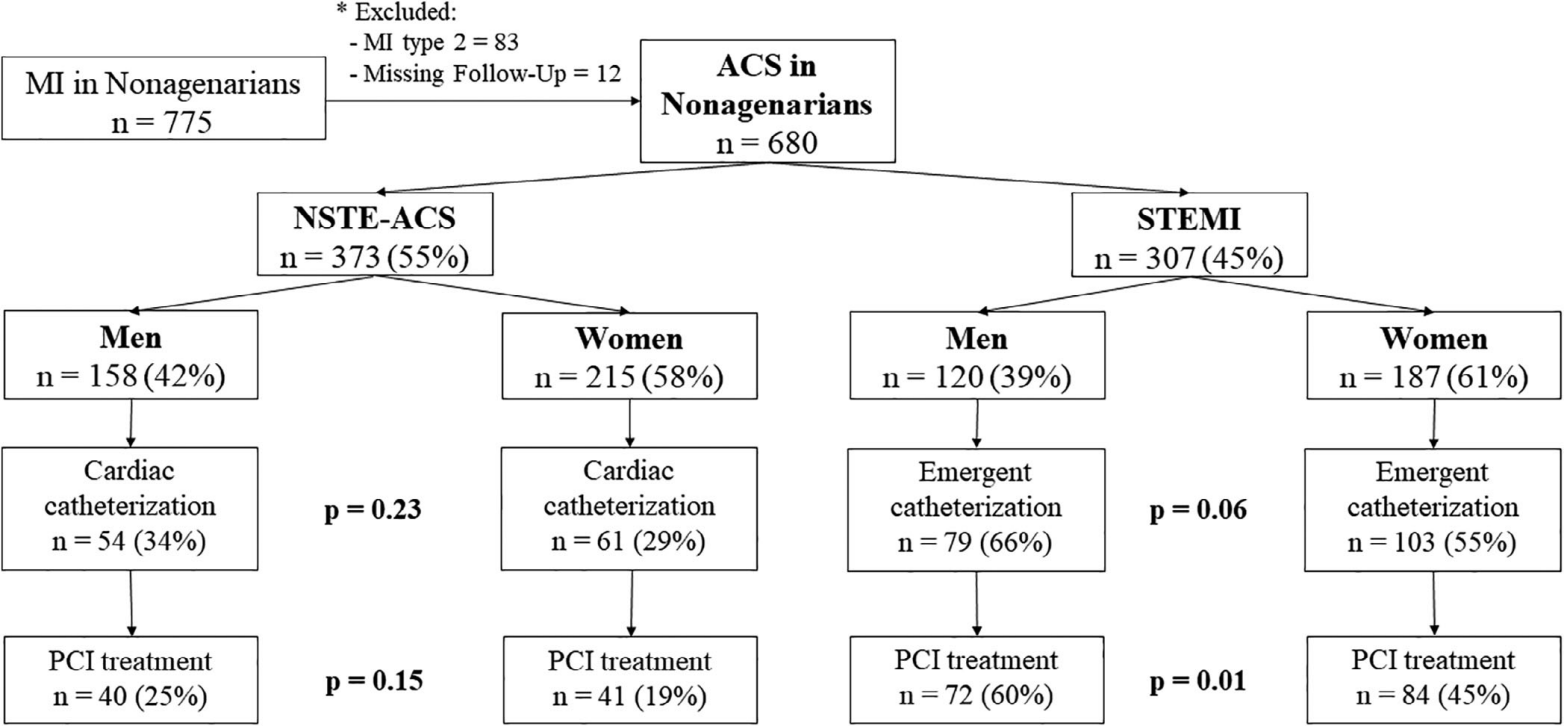
Title: Study Flowchart. Legend: From 2005 to 2018, 680 nonagenarian patients with acute coronary syndrome (ACS) were included. MI, myocardial infarction; STEMI, ST‐elevation myocardial infarction; NSTE‐ACS, non‐ST‐elevation acute coronary syndrome; PCI, percutaneous coronary intervention

This multicentre observational study included all consecutive patients aged ≥90 years who were hospitalized for ACS between 2005 and 2018 across four academic institutions. Only patients with type 1 myocardial infarction were included. The choice of treatment was based on the criteria of the attending medical staff. The flowchart of the study is presented in Figure 1. The follow‐up protocol included a review of medical records at 30 days and 1 year after hospital discharge. The four academic institutions are characterized by being tertiary and high complexity hospitals. Each serves a total catchment area of over half a million population and provides 24/7 ST‐segment elevation myocardial infarction (STEMI) percutaneous coronary intervention (PCI). The study was approved by the Ethics Committee and adhered to the principles outlined in the Declaration of Helsinki. All data were obtained by a retrospective review of cases using standardized report forms (Appendix S1). Standardized definitions of all patient‐related variables, clinical diagnoses, and hospital complications and outcomes were used. All patients admitted with an ACS were classified in STEMI or non‐ST‐segment elevation ACS (NSTE‐ACS), where unstable angina was included, according to current clinical practice guidelines^3^, ^4^ and fourth definition of myocardial infarction.^5^ The following outcomes were evaluated: mortality, recurrent myocardial infarction, stroke, and major bleeding. Cardiovascular mortality was defined as any death due to proximate cardiac cause (MI, low‐output failure, fatal arrhythmia), unwitnessed death, stroke, cardiovascular hemorrhage, death of unknown cause, and all procedure‐related deaths, including those related to concomitant treatment.^6^ Recurrent myocardial infarction was defined as any myocardial infarction according to the World Health Organization's extended definition.^7^ Stroke was defined as focal neurologic deficit lasting ≥24 hours or focal neurologic deficit lasting <24 hours with imaging findings of acute infarction or hemorrhage.^8^ Major bleeding was defined as Bleeding Academic Research Consortium score ≥ 3.^9^ The primary endpoint was sex‐based differences in ACS management. The second endpoints were 1‐year all‐cause mortality by sex. Long‐term survival was compared between patients undergoing PCI and those managed with medical treatment alone by sex.

Categorical variables are presented as frequencies (percentages), assessing the differences by *χ* ^2^ test (or Fisher test when necessary). Continuous variables are presented as a mean ± standard deviation or as a median (interquartile range). The Kolmogorov–Smirnov test was applied to ensure normal distribution. Continuous variables were compared using the analysis of variance test or the Kruskal–Wallis test, as appropriate. Survival curves were constructed for the time‐to‐event variables using the Kaplan–Meier method. Because differences in baseline characteristics could substantially interfere with the outcomes, propensity‐score matching was performed. Propensity score matching yielded 185 patients in the men group and 185 control subjects in the women group as Table S1. The balance between the two groups after propensity‐score matching was assessed by calculating percent standardized mean differences. Percent standardized mean differences after propensity‐score matching adjustment were within 15% across all matched covariates, demonstrating the achievement of successful balance between comparative groups. To identify independent predictors of PCIs by ACS and 1‐year all‐cause death in each group, we used a multivariable logistic and Cox proportional hazard regression, respectively. For all analyses, a two tailed *p* value <.05 was used as the criterion for statistical significance. Analyses were performed using STATA software (V 14.0, StataCorp LP, College Station, TX).

## 3 RESULTS

A total of 680 nonagenarian patients with an admission diagnosis of ACS were enrolled between January 2005 and December 2018. The diagnosis of STEMI was stablished in 307 (45%) whereas the number of patients with NSTE‐ACS was 373 (55%). Women were predominant in both groups (58% and 61%, respectively) as Figure 1 shows. The main baseline characteristics and clinical presentation of the two groups are presented in Table 1. Men presented a higher disease burden, including higher prevalence of peripheral artery disease (9% vs. 4%; *p* = .002), chronic obstructive pulmonary disease (19% vs. 4%; *p* < .001), active oncology disorder (11% vs. 4%; *p* = .006) and prior myocardial infarction (26% vs. 18%; *p* = .01) compared to women. Conversely, women were frailer with higher disability (5% vs. 11%; *p* < .001) and severe cognitive impairment (5% vs. 10%; *p* = .01).

**TABLE 1:** Baseline clinical characteristics

Variable	All patients (N = 680)	Men (n = 278)	Women (n = 402)	*p* value
Demographics				
Age (years)	92.6 ± 2.4	92.4 ± 2.2	92.6 ± 2.5	.35
Medical history				
Hypertension	541 (80)	208 (75)	333 (82)	.01
Hyperlipidemia	261 (38)	107 (39)	154 (38)	.96
Diabetes mellitus	203 (30)	82 (30)	121 (30)	.87
Chronic kidney disease	144 (21)	59 (21)	85 (21)	.98
Prior stroke				
Ischemic	93 (13)	38 (14)	55 (14)	.66
Hemorrhagic	3 (1)	2 (1)	1 (1)	
Peripheral artery disease	41 (6)	26 (9)	15 (4)	.002
Chronic obstructive pulmonary disease	71 (10)	54 (19)	17 (4)	<.001
Oncology disorders				
Previous	48 (7)	18 (7)	30 (8)	.006
Active	46 (7)	29 (10)	17 (4)	
Previous myocardial infarction	145 (21)	72 (26)	73 (18)	.01
Previous percutaneous coronary intervention	64 (9)	35 (13)	29 (7)	.01
Previous coronary artery bypass grafting	27 (4)	18 (7)	9 (2)	.005
Previous heart failure	111 (16)	47 (17)	64 (16)	.73
Frailty Characteristics				
Disability (activities of daily living)				
None	421 (62)	198 (74)	223 (57)	<.001
Semi‐independent	183 (28)	56 (21)	127 (32)	
Dependent	55 (8)	13 (5)	42 (11)	
Moderate or severe cognitive impairment	55 (8)	14 (5)	41 (10)	.01
Initial presentation				
Atypical symptoms	125 (18)	42 (15)	83 (21)	.07
Killip class at admission				
I	403 (59)	178 (64)	225 (56)	.09
II	184 (27)	71 (26)	113 (28)	
III	70 (11)	20 (7)	50 (12)	
IV	23 (3)	9 (3)	14 (3)	
GRACE score	173.4 ± 24	170.8 ± 22	175 ± 25	.03
CRUSADE score	46.5 ± 12	42.4 ± 10	49.8 ± 12	<.001
Serum creatinine at admission (mg/dl)	1.37 ± 0.67	1.50 ± 0.8	1.28 ± 0.6	<.001
Hemoglobin at admission (mg/dl)	12.2 ± 1.8	12.3 ± 1.9	12.1 ± 1.7	.08
Management approach				
Coronary angiogram	297 (44)	133 (48)	164 (41)	.07
PCI approach	237 (35)	112 (41)	125 (31)	.01

*Note:* Values are n (%) or mean ± SD.

Abbreviations: NSTE ACS, non‐ST‐elevation acute coronary syndrome; PCI, percutaneous coronary intervention; STEMI, ST‐elevation myocardial infarction.

“There was a non‐significant tendency to perform less emergent catheterization in women compared to men (48% vs. 41%; *p* = .07). These differences are mainly driven by the STEMI group (66% vs. 55%; *p* = .06). Regarding this, women were less likely than men to undergo a PCI (40% vs. 31%; *p* = .01), mainly in the STEMI group with a rate of subsequent PCI lower in women (60% vs. 45%; *p* = .01). These differences were not found in the NSTE‐ACS group as Figure 1 shows. Angiographic findings and interventional procedures are detailed in Table S2. No differences were found in catheterization access between groups, being the radial access predominant in both. There were no significant differences between the groups in terms of the TIMI flow grade III after the procedure (93% vs. 84%, *p* = .05). No differences were found in the medical treatment at discharge (Table S3).

**FIGURE 2:**
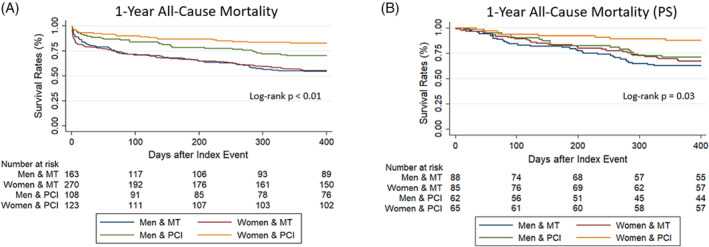
Title: Kaplan–Meier survival estimates for 1‐year All‐Cause Death. Legend by sex and treatment: 1‐year survival rates: All cohort (A) and propensity‐matching score's cohort (B). PCI, primary percutaneous coronary intervention. MT, medical treatment. PS, propensity‐matching score

A comparison of clinical outcomes between the women and men groups is presented in Table 2 and Figure 2. At 1‐year follow‐up, a total of 263 patients had died (39%). Overall mortality rates in men and women were similar both in‐hospital and at 1‐year follow‐up (16% vs. 18%; *p* = .4 and 41% vs. 37%; *p* = .3, respectively). However, patients treated with PCI showed better survival rates than those managed with medical therapy alone. Furthermore, PCI survival benefit at 1‐year was greater in women compared to their male counterparts (82% vs. 68%; *p* = .008; Figure 2(A)), persisting after sensitivity analyses using propensity‐score matching (80% vs. 64%; *p* = .03; Figure 2(B)). Female sex remains in the multivariate logistic regression analysis (Table 3) as an independent predictor for not undergoing a PCI in STEMI (OR: 0.62; 95% CI: 0.38–0.97; *p* = .03) along with age (OR: 0.88; 95% CI: 0.79–0.98; *p* = .02). The absence of disability was related to undergoing PCI in STEMI (OR: 1.82; 95% CI: 1.03–3.22; *p* = .04) as well as in NSTE‐ACS (OR: 3.34; 95% CI: 1.83–6.08; *p* < .001). On the other hand, in multivariate Cox proportional hazard models (Table S4), PCI was independently associated with a lower risk of 1‐year all‐cause death in both groups (HR men: 0.63; 95% CI: 0.41–0.96; *p* = .03; HR women: 0.37; 95% CI: 0.23–0.60; *p* < .001).

**TABLE 2:** Outcomes

Variable	All patients (N = 680)	Men (n = 278)	Women (n = 402)	*p* value
In‐hospital				
Death	115 (17)	43 (16)	72 (18)	.40
Major or clinically relevant bleeding	10 (2)	8 (3)	2 (1)	.01
Acute kidney injury stage 3	45 (7)	18 (7)	27 (7)	.90
Mechanical complications^a^	22 (3)	9 (3)	13 (3)	.99
Left ventricular ejection fraction ≤30%	84 (12)	35 (13)	49 (12)	.88
1‐year follow‐up				
All‐cause death	263 (39)	113 (41)	150 (37)	.38
Cardiovascular death	162 (24)	63 (12)	97 (24)	.18
Recurrent MI	73 (11)	33 (12)	40 (10)	.41
Stroke	25 (4)	9 (3)	16 (4)	.59
Major or clinically relevant bleeding	17 (3)	9 (3)	8 (2)	.30
Rehospitalization by heart failure	127 (19)	49 (18)	78 (19)	.86

*Note:* Values are n (%) or mean ± SD.

Abbreviations: MI, myocardial infarction; NSTE ACS, non‐ST‐elevation acute coronary syndrome; PCI, percutaneous coronary intervention; STEMI, ST‐elevation myocardial infarction.

^a^ Ventricular septal rupture, free wall rupture and ischemic mitral regurgitation by papillary muscle rupture.

**TABLE 3:** Independent predictors for percutaneous coronary intervention by ACS

Characteristic	Univariate analysis		Multivariate analysis	
	OR (95% CI)	*p* value	OR (95% CI)	*p* value
Non‐ST‐elevation acute coronary syndrome				
Women	0.70 (0.43–1.14)	0.15	‐	‐
Age	0.90 (0.80–1.01)	0.08	‐	‐
Chronic obstructive lung disease	1.02 (0.46–2.23)	0.97	‐	‐
Chronic kidney disease	3.92 (2.03–7.54)	< 0.001	‐	‐
Peripheral vascular disease	1.48 (0.55–3.94)	0.44	‐	‐
Active oncology disorder	0.87 (0.34–2.20)	0.77	‐	‐
No disability for activities of daily living	3.33 (1.86–5.96)	< 0.001	3.34 (1.83–6.08)	< 0.001
Moderate or severe cognitive impairment	0.45 (0.25–0.80)	0.006	‐	‐
Atypical symptoms	0.32 (0.14–0.72)	0.006	‐	‐
Killip class >2 at admission	0.51 (0.22–1.17)	0.11	‐	‐
Institutions			‐	‐
A	Ref.	Ref.		
B	0.53 (0.19–1.7)	0.19		
C	1.47 (0.82–2.66)	0.21		
D	1.26 (0.59–2.80)	0.57		
ST‐elevation myocardial infarction				
Women	0.54 (0.34–0.87)	0.01	0.62 (0.38–0.97)	0.03
Age	0.86 (0.77–0.95)	0.002	0.88 (0.79–0.98)	0.02
Chronic Kidney Disease	0.79 (0.62–1.00)	0.05	‐	‐
Peripheral vascular disease	0.39 (0.15–1.05)	0.06	‐	‐
Chronic Obstructive Lung Disease	1.12 (0.53–2.38)	0.77	‐	‐
Active oncology disorder	0.64 (0.23–1.72)	0.37	‐	‐
No disability for activities of daily living	2.63 (1.61–4.30)	< 0.001	1.82 (1.03–3.22)	0.04
Moderate or severe cognitive impairment	0.50 (0.31–0.76)	0.002	‐	‐
Atypical symptoms	0.63 (0.34–1.15)	0.134	‐	‐
Killip class >2 at admission	0.61 (0.31–1.19)	0.145	‐	‐
Institutions			‐	‐
A	Ref.	Ref.		
B	1.47 (0.82–2.66)	0.19		
C	1.77 (0.88–2.93)	0.23		
D	2.10 (0.97–4.55)	0.06		

*Note:* Odd ratios and their 95% confidence intervals were calculated by multivariate logistic regression analysis.

Abbreviation: ACS, acute coronary syndrome.

## 4 DISCUSSION

There are two main findings of our study. First, nonagenarian women were less likely to undergo an emergent catheterization compared to men, mainly in a STEMI context. Second, coronary revascularization in women was associated with a lower risk of 1‐year all‐cause mortality compared to men even after sensitivity analysis.

Cardiovascular disease is the leading cause of death in women in developed countries and its incidence is increasing in developing countries, with ischemic heart disease being the main etiology.^10^ Despite the proven efficacy of coronary revascularization, many studies have shown that women are less likely to be referred for revascularization in ACS in different contexts: chronic coronary syndrome, NSTE‐ACS, and STEMI.^11^ On the other hand, older patients and women are underrepresented in contemporary ACS trials, since the development of observational studies is necessary to measure the real impact of ACS in this subgroup.^12^ In our study, female nonagenarians admitted for ACS were treated significantly less often with an invasive approach compared to men, leading to lower revascularization rates, which is significant in the STEMI group. This sex‐gender disparity in ACS has been previously described mainly in young populations, as shown by the study VIRGO (*The Variation in Recovery: Role of Gender on Outcomes of Young AMI Patients*) where young women with STEMI were less likely to receive reperfusion therapy and more likely to have reperfusion delays than similarly aged men. Similar results have been observed in women with NSTE‐ACS^13^ and chronic coronary syndrome.^14^ We evidenced the same pattern in nonagenarians despite the fact that PCI appeared to be beneficial in women. Sulzgruber et al.^15^ showed that elderly women (≥80 years) with ACS obtained a higher benefit of any coronary intervention in cardiovascular mortality rate compared to men. Hao et al. evaluated sex differences in acute management, medical therapies for secondary prevention, and in‐hospital mortality in 82 196 patients admitted for ACS at 192 hospitals in China, using data from the *Improving Care for Cardiovascular Disease in China‐Acute Coronary Syndrome* project (CCC‐ACS).^16^ They found that women were less likely to receive evidence‐based acute treatments for ACS than men, including early dual antiplatelet therapy, heparins during hospitalization, and reperfusion therapy for ST‐segment‐elevation myocardial infarction and fewer strategies for secondary prevention after hospital discharge. Similar results were found by Hvelplund et al.^17^ In a cohort of 9561 women and 16 406 men, significantly fewer women underwent coronary angiogram (cumulative incidence 64% for women vs. 78% for men, *p* < .05) compared with men. Subsequently, revascularization was also less likely in women compared to men (HR = 0.68, 95% CI = 0.66–0.71, *p* < .0001). This gender gap in optimal medical treatment is also observed in other clinical conditions such as atrial fibrillation.^18^ At this point it is very important to underline that ACS guidelines highlight that primary PCI is recommended in STEMI regardless of age and sex.^3^ To the best of our knowledge, our study is the first where these sex‐gender differences are evaluated in a fast‐growing and predominantly female population, such as nonagenarians.

A higher burden of frailty characteristics observed in women may influence physicians to adopt less invasive strategies. Subsequently, attending medical staff may have selected a non‐invasive approach due to less anticipated life expectancy. In our study, frailty, measured as the presence of disability and/or cognitive dysfunction, could influence clinical decisions in women. It has been broadly reported that women at any age have a higher frailty index compared to men. Nevertheless, their life expectancy used to be longer than men. This inverse association between health and longevity is known as the morbidity–mortality paradox.^19^ This phenomenon is not fully understood, but hypotheses include higher self‐reported deficits,^20^ more diseases that affect quality of life rather than mortality, and higher physiological reserve in women compared to men.^21^ Psychosocial factors, such as healthcare utilization and self‐reported behaviors have also been implicated in the morbidity‐mortality paradox.^22^ On the other hand, another possible explanation for the lower intention of coronary intervention in women could be a higher rate of understanding of postprocedural complications in this group. Several studies have demonstrated a higher rate of in‐hospital complications and higher rates of bleeding in women, but these sex differences disappear after adjusting for different comorbidities^13^ and the use of appropriate antithrombotic treatment and vascular access.^23^ Awareness of cardiovascular disease (CVD) among women has improved over the past 15 years, but awareness of atypical symptoms remains low.^24^ This might be conducive to misdiagnosis, resulting in a delay in receiving appropriate acute treatments for ACS, including a coronary angiogram and subsequent PCI if indicated. This conservative approach in the female group compared to men could be explained by the wrong perception that ischaemic cardiac symptoms, often less specific in women, are related to other pathologies.^25^ The use of a systems‐based approach to STEMI care may help reduce sex disparities and improves STEMI care and outcomes in women, regardless of age.^26^

## 5 STUDY LIMITATIONS

The main limitation of this study is its observational design, which implies an inherent selection bias. Moreover, it is difficult to capture and control all potential confounders when using a registry. Despite the use of a propensity‐matching score, we cannot control for all potential confounders when using data from a registry. Therefore, the purpose of the study is exploratory, and our results should be considered as hypothesis‐generating. In addition, the sample size may lack the power to detect other statistically significant differences in outcomes between groups. Also, we lacked data regarding cardiac rehabilitation reference at hospital discharge and during follow‐up. Therefore, we cannot compare cardiac rehabilitation reference rates at hospital discharge between groups.

## 6 CONCLUSION

In conclusion, sex‐gender disparities have been observed in nonagenarians. Despite receiving less often invasive approaches, women showed better clinical outcomes. This finding may help increase awareness and reduce the current gender gap in ACS management at any age.

## CONFLICT OF INTEREST

This research did not receive any specific grant from funding agencies in the public, commercial, or not‐for‐profit sectors.

## AUTHOR CONTRIBUTION

Pedro L. Cepas‐Guille takes responsibility for all aspects of the reliability and freedom from bias of the data presented and their discussed interpretation.

## Supporting information


**Appendix** S1. Supporting Information.

**TABLE S1.** Percent standardized differences of variables among unadjusted and propensity‐score matched, and baseline characteristics of propensity‐score matched cohort.

**TABLE S2.** Baseline Lesion‐ and Procedure‐Related Profiles.

**TABLE S3.** A, Treatment at hospital discharge in NST‐ACS. B, Treatment at hospital discharge in STEMI.

**TABLE S4.** Independent predictors for 1‐year all‐cause death by sex.
