# Supplementary material for: Sex‐gender disparities in nonagenarians with acute coronary syndrome
Source: Clin Cardiol. 2021 Jan 19;44(3):371–8. doi: 10.1002/clc.23545 (PMC7943909; doi:10.1002/clc.23545)
Supplement: Supplementary file 1 — Appendix S1. Supporting Information. [file CLC-44-371-s005.docx]

| Hospital: Clinic □ Clínico □ León □ Mar □ | Medical Record Number ……………………………. |
| --- | --- |
| Event date (dd/mm/yyyy) ........................................... | Discharge date (hh:mm) ............................................... |
| Birth date (dd/mm/yyyy) ................................  Age ………………………………… | Admission unit: CCU □ ICCU □ Cardiology Ward □ |
| Gender: Female □ Male □ | Weight …………………. Height ……………….. |

**Filiation:**

**Medical history:**

| **Risk factors** |  | |  | |  | |  | |
| --- | --- | --- | --- | --- | --- | --- | --- | --- |
| Diabetes □ | HTA □ | Hyperlipidemia □ | | CV family history □ | | | |  |
| Alcohol □ | Chronic kidney disease □ | | Smoker: active □ former □ no□ | | |  | | |
| **CV disease** |  | |  | |  | |  | |
| PCI □ | Myocardial infarction □ | | Peripheral artery disease □ | | |  | | |
| CABG □ | Heart failure □ | | Stroke  ischemic □ hemorrhagic □ | | |  | | |
| **Others** | COPD □ | | Neoplasia: prior □ active □ no □ | | |  | | |
|  | Charlson score ……………….. | |  |  |  |  |  |  |

**Previous treatment:**

| ASA □ | P2Y12 inhibitor □ | Oral anticoagulation □ | Statins □ |
| --- | --- | --- | --- |

**Frailty characteristics:**

| **Disability** |  |  | | |  | | | |  | |
| --- | --- | --- | --- | --- | --- | --- | --- | --- | --- | --- |
| None □ | Semi-independent □ | | Dependent □ | | | | Barthel score ……….. | | |  |
| **Cognitive impairment** |  | | |  | |  | | | | |
| Mild □ | Moderate □ | Severe □ | | | | | |  | | |

**Clinical presentation**

| **Chest pain** | Typical □ | | Atypical □ | |  |  |  |  |
| --- | --- | --- | --- | --- | --- | --- | --- | --- |
| **EKG changes** | No □ | | Non-specific □ | | Subendocardial □ | Subepicardial □ | | |
| **STEMI localization** | Anterior □ | | Inferior □ | | Lateral □ | Posterior □ | | |
| **Killip class** | I □ | | II □ | | III □ | IV □ | | |
| Blood pressure ……………… | |  | | Heart rate ……………………. | | |  |  |
| GRACE score …………….. | |  | | CRUSADE score ……………. | | |  |  |

**Blood tests**:

| Hb ................... | Hematocrit……………………….. |
| --- | --- |
| cTn ..................... | CK…………................... |
| Cholesterol ...................... | LDL ............. HDL ................ |
| Creatinine............................. | GFR………………..................... |

**Management approach:**

| Coronariography □ Urgent □ Deferred □ no□ | | | | |
| --- | --- | --- | --- | --- |
| Ischemia test □ | Treadmill □ | SPECT □ | Echo □ | CMR □ |
| TTE □ | EF (%) …………….. |  |  |  |

**Catheterization procedure:**

| **Access** | Radial □ | | Femoral □ |  |  |  |  |  |
| --- | --- | --- | --- | --- | --- | --- | --- | --- |
| **Culprit artery** | LAD □ | | Diagonal □ | Circumflex □ | Obtuse marginal □ | | |  |
|  | RCA □ | | PDA □ | Right marginal □ | Other □ | | |  |
| **LMCA affection** | Yes □ | | No □ |  |  |  |  |  |
| **TIMI flow before PCI** | 0 □ | | 1 □ | 2 □ | 3 □ | | | |
| **Number of vessels** | Single □ | | Two □ | Three □ |  |  |  |  |
| **PCI** | Yes □ | | No □ |  |  |  |  |  |
| **Stents** | BMS □ | | DES □ |  |  |  |  |  |
| **TIMI flow post PCI** | 0 □ | | 1 □ | 2 □ | 3 □ | | | |
| **Complete revascularization** | |  | Yes □ No □ | | |  |  |  |

**Treatment at hospital discharge:**

| ASA □ | Vit K Antagonist □ |
| --- | --- |
| Clopidogrel □ | NOACs □ |
| Ticagrelor □ | Statins □ |
| PPI □ | Beta-blockers □ |
| ACEi or ARB □ | Aldosterone inhibitors □ |
| Amiodarone □ |  |

**In-hospital outcomes:**

| **Death □** | Date of death (hh:mm) ............................................ |
| --- | --- |
| **Bleeding □** | Mayor □ Minor □ |
| **Acute kidney injury □** | I □ II □ III □ IV □ V □ |
| **Acute care** □ | Vasoactive drugs □  Temporal pacemaker □  Mechanical ventilation □ Invasive □ Non-invasive □  IABP □ |
| **Arrhythmic complications** □ | VT □ VFib □ AV block □ |
| **Mechanical complications □** | Free wall rupture □  Ventricular septal rupture □  MR by papillary muscle rupture □ |

**1-year follow-up outcomes:**

| **Death** □ CV death □ Non-CV death □  Date (dd/mm/yyyy) ........................................... | **Myocardial infarction** □ |
| --- | --- |
| **Stroke** □ | **Bleeding**: Mayor □ Minor □ No □ |
| **Heart failure hospitalization** □ |  |
