# Supplementary material for: Sex‐gender disparities in nonagenarians with acute coronary syndrome
Source: Clin Cardiol. 2021 Jan 19;44(3):371–8. doi: 10.1002/clc.23545 (PMC7943909; doi:10.1002/clc.23545)
Supplement: Supplementary file 2 — TABLE S1. Percent standardized differences of variables among unadjusted and propensity‐score matched, and baseline characteristics of propensity‐score matched cohort. [file CLC-44-371-s002.docx]

| Table S1 Percent standardized differences of variables among unadjusted and propensity-score matched, and baseline characteristics of propensity-score matched cohort. | | | | | |
| --- | --- | --- | --- | --- | --- |
| **Variable** | **Percent standardized differences** | | **Propensity-score matched cohort (N = 370)** | | |
|  | **Unadjusted**  **(N = 680)** | **PS matched**  **(N = 370)** | **Men**  **(N = 185)** | **Women**  **(N = 185)** | **P value** |
| Age (years) | -0.0738 | 0.0685 | 92.31 ± 2.2 | 92. ± 2.26 | 0.51 |
| Oncology disorders   - Previous - Active | 0.0762 | 0.0620 | 13 (7)  12 (7) | 9 (5)  14 (8) | 0.64 |
| Chronic obstructive pulmonary disease | 0.4833 | -0.0622 | 12 (7) | 15 (8) | 0.55 |
| Peripheral artery disease | 0.2284 | 0.0622 | 15 (8) | 12 (7) | 0.55 |
| Disability (activities of daily living):   - None - Semi-independent - Dependent | 0.3784 | 0.1158 | 131 (71)  42 (23)  12 (7) | 139 (75)  38 (21)  8 (4) | 0.54 |
| Moderate or severe cognitive impairment | -0.1978 | 0.1004 | 11 (6) | 7 (4) | 0.33 |
| Previous myocardial infarction | 0.1873 | 0.0809 | 40 (22) | 34 (18) | 0.44 |
| Previous coronary artery bypass grafting | 0.2082 | 0.0000 | 8 (4) | 8 (4) | 1.00 |
| Killip class at admission:   - I - II - III - IV | 0.2047 | 0.0000 | 115 (62)  49 (27)  15 (8)  6 (3) | 115 (62)  49 (27)  15 (8)  6 (3) | 1.00 |
| Serum creatinine at admission (mg/dL) | 0.3208 | -0.0028 | 1.42 ± 0.6 | 1.42 ± 0.7 | 0.98 |
| Hemoglobin at admission (mg/dL) | -0.1607 | -0.0110 | 12.3 ± 1.9 | 12.3 ± 1.6 | 0.93 |
| Values are n (%) or mean ± SD. PS, propensity score | | | | | |
