# Supplementary material for: Sex‐gender disparities in nonagenarians with acute coronary syndrome
Source: Clin Cardiol. 2021 Jan 19;44(3):371–8. doi: 10.1002/clc.23545 (PMC7943909; doi:10.1002/clc.23545)
Supplement: Supplementary file 3 — TABLE S2. Baseline Lesion‐ and Procedure‐Related Profiles. [file CLC-44-371-s003.docx]

| **TABLE S2 Baseline Lesion- and Procedure-Related Profiles** | | | | |
| --- | --- | --- | --- | --- |
| **Variable** | **All patients**  **(N = 297)** | **Men**  **(n = 115)** | **Women**  **(n = 182)** | **P value** |
| Catheterization access   - Radial access - Femoral access | 198 (67)  99 (31) | 85 (64)  48 (36) | 113 (69)  51 (31) | 0.36 |
| Infarct-related coronary artery   - Left anterior descending artery - Diagonal coronary artery - Circumflex coronary artery - Obtuse marginal artery - Right coronary artery - Posterior descending artery - Right marginal coronary artery | 146 (50)  6 (3)  28 (10)  9 (4)  81 (28)  5 (2)  5 (2) | 68 (53)  3 (2)  14 (11)  5 (4)  33 (27)  2 (2)  1 (1) | 78 (51)  3 (2)  14 (9)  4 (3)  48 (31)  3 (2)  4 (3) | 0.83 |
| Left main coronary artery (non-culprit) | 32 (11) | 19 (14) | 13 (8) | 0.10 |
| TIMI flow grade before PCI   - 0 - 1 - 2 - 3 | 123 (42)  32 (11)  38 (13)  104 (35) | 53 (40)  15 (11)  19 (14)  45 (34) | 70 (43)  17 (10)  19 (12)  58 (35) | 0.88 |
| Coronary arteries affected   - Single-vessel coronary disease - Two-vessel coronary disease - Three-vessel coronary disease | 127 (43)  99 (33)  71 (24) | 47 (36)  52 (39)  33 (25) | 79 (49)  45 (28)  36 (23) | 0.05 |
| PCI performed | 237 (80) | 112 (84) | 125 (76) | 0.08 |
| Coronary stenting   - BMS - DES | 122 (52)  115 (49) | 58 (52)  54 (48) | 63 (51)  61 (49) |  |
| TIMI flow grade after procedure:   - 0 - 1 - 2 - 3 | 18 (6)  5 (2)  10 (4)  261 (88) | 4 (3)  3 (2)  2 (2)  118 (93) | 13 (8)  2 (1)  10 (6)  134 (84) | 0.05 |
| Complete revascularization | 105 (33) | 43 (32) | 62 (38) | 0.33 |
| PCI periprocedural complications | 3 (1) | 2 (2) | 1 (1) | 0.56 |
| Values are n (%). PCI = percutaneous coronary intervention; STEMI = ST-elevation myocardial infarction; NSTE-ACS = non-ST-elevation acute coronary syndrome; BMS = bare metal stent; DES = drug-eluting stent; TIMI = thrombolysis in myocardial infarction | | | | |
