# Supplementary material for: Sex‐gender disparities in nonagenarians with acute coronary syndrome
Source: Clin Cardiol. 2021 Jan 19;44(3):371–8. doi: 10.1002/clc.23545 (PMC7943909; doi:10.1002/clc.23545)
Supplement: Supplementary file 4 — TABLE S3. A, Treatment at hospital discharge in NST‐ACS. B, Treatment at hospital discharge in STEMI. [file CLC-44-371-s004.docx]

| Supplementary Table 3A **Treatment at hospital discharge in NST-ACS** | | | | |
| --- | --- | --- | --- | --- |
|  | **All patients**  **(n = 315)** | **Men**  **(n = 134)** | **Women**  **(n = 181)** | **p value** |
| Aspirin | 259 (82) | 108 (81) | 151 (83) | 0.52 |
| Clopidogrel | 154 (49) | 68 (51) | 86 (48) | 0.57 |
| Ticagrelor | 2 (1) | 1 (1) | 1 (1) | 0.87 |
| Vitamin K antagonist | 41 (13) | 17 (13) | 24 (13) | 0.87 |
| NOACs | 14 (5) | 7 (5) | 7 (4) | 0.57 |
| PPI | 267 (85) | 115 (86) | 152 (85) | 0.82 |
| Beta-blockers | 187 (60) | 75 (56) | 112 (63) | 0.27 |
| Statins | 225 (72) | 98 (73) | 127 (71) | 0.67 |
| ACEI or ARB | 155 (50) | 54 (40) | 101 (56) | 0.008 |
| Aldosterone inhibitors | 19 (6) | 10 (7) | 9 (5) | 0.37 |
| Amiodarone | 11 (4) | 5 (4) | 6 (3) | 0.85 |
| Supplementary Table 3B **Treatment at hospital discharge in STEMI** | | | | |
|  | **All patients**  **(n = 237)** | **Men**  **(n = 95)** | **Women**  **(n = 142)** | **p value** |
| Aspirin | 221 (93) | 89 (94) | 132 (93) | 0.83 |
| Clopidogrel | 164 (70) | 72 (76) | 92 (62) | 0.09 |
| Ticagrelor | 3 (1) | 0 | 3 (2) | 0.15 |
| Vitamin K antagonist | 23 (10) | 10 (11) | 13 (9) | 0.73 |
| NOACs | 12 (5) | 5 (5) | 7 (5) | 0.46 |
| PPI | 209 (88) | 79 (84) | 130 (91) | 0.11 |
| Beta-blockers | 141 (60) | 57 (61) | 84 (59) | 0.82 |
| Statins | 194 (82) | 77 (82) | 117 (82) | 0.93 |
| ACEI or ARB | 133 (56) | 55 (59) | 78 (55) | 0.59 |
| Aldosterone inhibitors | 22 (10) | 10 (11) | 12 (9) | 0.56 |
| Amiodarone | 12 (5) | 5 (5) | 7 (5) | 0.89 |
| Values are n (%). PCI, primary percutaneous coronary intervention; NOACs, Non-vitamin K antagonist oral anticoagulants; PPI, proton pump inhibitors; ACEI, angiotensin converting enzyme inhibitors; ARB, angiotensin receptors blocker. | | | | |
