# Supplementary material for: Sex‐gender disparities in nonagenarians with acute coronary syndrome
Source: Clin Cardiol. 2021 Jan 19;44(3):371–8. doi: 10.1002/clc.23545 (PMC7943909; doi:10.1002/clc.23545)
Supplement: Supplementary file 5 — TABLE S4. Independent predictors for 1‐year all‐cause death by sex. [file CLC-44-371-s001.docx]

| **TABLE S4 Independent predictors for 1-year all-cause death by sex** | | | | |
| --- | --- | --- | --- | --- |
| **Characteristic** | **Univariate Analysis** | | **Multivariate Analysis** | |
|  | **HR (95% CI)** | **p Value** | **HR (95% CI)** | **p Value** |
| **Men** | | | | |
| Age | 1.05 (0.97-1.14) | 0.19 | - | - |
| Diabetes | 1.01 (0.67-1.54) | 0.96 | - | - |
| Serum creatinine at admission | 1.36 (1.11-1.68) | 0.003 | - | - |
| Peripheral artery disease | 1.87 (1.07-3.28) | 0.03 | 2.02 (1.15 – 3.57) | 0.02 |
| Active oncology disorder | 1.11 (0.61-2.03) | 0.74 | - | - |
| No disability | 0.63 (0.42 – 0.96) | 0.03 | - | - |
| Moderate or severe cognitive impairment | 1.18 (0.87 – 1.60) | 0.28 | - | - |
| Killip class > 2 | 3.05 (1.85 – 5.02) | <0.001 | 3.06 (1.83 – 5.12) | <0.001 |
| Left ventricular ejection fraction ≤30% | 1.19 (0.69 – 2.06) | 0.53 | - | - |
| Percutaneous coronary intervention | 0.57 (0.38 – 0.87) | 0.009 | 0.63 (0.41 – 0.96) | 0.03 |
| STEMI | 1.18 (0.81 – 1.73) | 0.39 | - | - |
| **Women** | | | | |
| Age | 1.04 (0.97-1.11) | 0.27 | - | - |
| Diabetes | 1.23 (0.86-1.75) | 0.25 | - | - |
| Serum creatinine at admission | 2.07 (1.68-2.54) | <0.001 | 1.87 (1.53 – 2.30) | <0.001 |
| Peripheral artery disease | 0.92 (0.38-2.26) | 0.86 | - | - |
| Active oncology disorder | 1.83 (0.96-3.48) | 0.07 | - | - |
| No disability | 0.54 (0.39-0.76) | <0.001 | - | - |
| Moderate or severe cognitive impairment | 1.47 (1.22-1.77) | <0.001 | 2.13 (1.35 – 3.34) | 0.001 |
| Killip class > 2 | 2.45 (1.65 – 3.64) | <0.001 | 2.01 (1.32 – 3.01) | 0.001 |
| Left ventricular ejection fraction ≤30% | 1.35 (0.85 – 2.15) | 0.20 | - | - |
| Percutaneous coronary intervention | 0.32 (0.20 – 0.51) | <0.001 | 0.37 (0.23 – 0.60) | <0.001 |
| STEMI | 0.82 (0.59 – 1.15) | 0.25 | - | - |
| Hazard ratios and their 95% confidence intervals were calculated by multivariable Cox regression analysis. HR = odds ratio; CI = confidence interval; STEMI = ST-segment elevation myocardial infarction | | | | |
